# Supratotal Surgical Resection for Low-Grade Glioma: A Systematic Review

**DOI:** 10.3390/cancers15092493

**Published:** 2023-04-26

**Authors:** Daniel Kreatsoulas, Mark Damante, Maxwell Gruber, Olivia Duru, James Bradley Elder

**Affiliations:** 1 Department of Neurological Surgery, The Ohio State University Wexner Medical Center, Columbus, OH 43210, USA; daniel.kreatsoulas@osumc.edu (D.K.);; 2 College of Medicine, The Ohio State University, Columbus, OH 43210, USA

**Keywords:** supratotal, low-grade glioma, safety, feasibility, outcomes

## Abstract

**Simple Summary:**

Low-grade gliomas are slow-growing, progressive tumors of the brain that invariably become high grade. They present a challenging entity because they can invade normal brain without many changes on radiologic scans. Standard treatment involves maximal safe removal via surgery, then close monitoring or other treatments, depending on whether portions were left. Some authors recommend removing a larger area of the brain than can be seen as tumor on imaging (called supratotal resection) because it theoretically gives patients a potential for longer disease-free survival. However, removing the adjacent “normal” brain carries the risk of neurological harm, which has tempered widespread adoption of the supratotal technique in lieu of preserving patients’ function. In this review, literature surrounding supratotal resection is explored systematically, and while there are no randomized trials, some evidence may suggest that supratotal resection is safe and effective as standard resection. Further studies are required to fully answer this question.

**Abstract:**

Low-grade gliomas (LGGs) are optimally treated with up-front maximal safe surgical resection, typically defined as maximizing the extent of tumor resection while minimizing neurologic risks of surgery. Supratotal resection of LGG may improve outcomes beyond gross total resection by removing tumor cells invading beyond the tumor border as defined on MRI. However, the evidence regarding supratotal resection of LGG, in terms of impact on clinical outcomes, such as overall survival and neurologic morbidities, remains unclear. Authors independently searched the PubMed, Medline, Ovid, CENTRAL (Cochrane Central Register of Controlled Trials), and Google Scholar databases for studies evaluating overall survival, time to progression, seizure outcomes, and postoperative neurologic and medical complications of supratotal resection/FLAIRectomy of WHO-defined LGGs. Papers in languages other than English, lacking full-text availability, evaluating supratotal resection of WHO-defined high-grade gliomas only, and nonhuman studies were excluded. After literature search, reference screening, and initial exclusions, 65 studies were screened for relevancy, of which 23 were evaluated via full-text review, and 10 were ultimately included in the final evidence review. Studies were evaluated for quality using the MINORS criteria. After data extraction, a total of 1301 LGG patients were included in the analysis, with 377 (29.0%) undergoing supratotal resection. The main measured outcomes were extent of resection, pre- and postoperative neurological deficits, seizure control, adjuvant treatment, neuropsychological outcomes, ability to return to work, progression-free survival, and overall survival. Overall, low- to moderate-quality evidence was supportive of aggressive, functional boundary-based resection of LGGs due to improvements in progression-free survival and seizure control. The published literature provides a moderate amount of low-quality evidence supporting supratotal surgical resection along functional boundaries for low-grade glioma. Among patients included in this analysis, the occurrence of postoperative neurological deficits was low, and nearly all patients recovered within 3 to 6 months after surgery. Notably, the surgical centers represented in this analysis have significant experience in glioma surgery in general, and supratotal resection specifically. In this setting, supratotal surgical resection along functional boundaries appears to be appropriate for both symptomatic and asymptomatic low-grade glioma patients. Larger clinical studies are needed to better define the role of supratotal resection in LGG.

## 1. Introduction

Low-grade gliomas (LGGs) are primary central nervous system (CNS) tumors, which account for around 10% of intracranial neoplasms in young adults [1]. LGGs are known to be slowly progressive but invariably transform to high-grade gliomas (HGGs), including glioblastoma (GBM). The median survival for LGG is between 5 and 10 years [2], and the goal of treatment is to preserve quality of life while improving progression-free (PFS) and overall survival (OS) [2,3]. Due to variations in tumor size and anatomic location, as well as patient variables, such as medical comorbidities and age, surgical and oncological treatment strategies can vary [2,4,5]. The standard initial treatment remains cytoreductive surgery, with the goal of gross total resection (GTR), followed by chemotherapy and/or radiation for high-risk patients (i.e., those with incomplete resection, older than 40 at diagnosis, and/or with IDH-wild-type status) [1,6].

For patients with glioma, including LGG and HGG, the extent of resection (EOR) appears to play a role in survival. EOR in LGG is defined as percent of removal of the FLAIR signal. EOR in HGG is defined as percent removal of enhancing tissue and necrotic center. Studies evaluating the extent of resection in LGG have shown that achieving GTR allows younger patients to be considered low risk relative to patients with partial resection and improves survival [2,3,4]. Similarly, EOR studies in HGG have shown a correlation of survival with EOR assuming no neurologic morbidity of surgery [7,8,9,10]. Most of these studies considered 100% removal to be a gross total resection and the maximum goal of surgery.

More recently, clinical studies have investigated the role of supratotal resection (SupTR) in glioma [11]. In HGG, this is defined as GTR plus resection of the FLAIR signal surrounding the enhancing and necrotic tumor mass. For LGG, this is defined as GTR of the FLAIR signal and additional removal of radiographically normal brain surrounding the tumor. In many studies regarding surgery for LGG, supratotal resection is confirmed on the postoperative MRI at 3 months by finding that the resection cavity is larger than the original FLAIR volume. The justification for supratotal resection is to achieve removal of invading cells near the radiographic margin of the tumor when neurologic risks of doing so are low [12,13,14]. When investigated in clinical case series of HGG and GBM, the evidence is mixed due to the suggestion of increased risk of deteriorating neurological function when performing SupTR, in spite of the suggestion of improved PFS and OS [11,15]. This concept has also been investigated in laser interstitial thermal therapy (LITT) of brain tumors. Recent studies of LITT have demonstrated that a higher extent of ablation, including supratotal ablation, leads to improved progression-free survival (PFS) and overall survival (OS) in HGG, mirroring the literature on extent of open resection [9,16,17].

The primary risk of supratotal resection is neurologic morbidity. Gliomas often are located in or near eloquent areas of the brain, and supratotal resection may lead to functional deficits. Surgeons utilizing functional boundary-centered resection techniques, such as white matter mapping, cortical and subcortical stimulation, and phase reversal, can remove tissue that may be outside the bounds of the FLAIR abnormalities while simultaneously preserving the patient’s function, further improving the degree of cytoreduction [13]. Additional imaging techniques and surgical adjuncts, such as intraoperative MRI, functional MRI, and awake mapping, can help increase the potential for supratotal resection. However, the clinical benefit of supratotal resection remains unclear.

With the concerns of functional deficits after surgery balanced by the potential for improved OS and PFS, supratotal resection has not been studied in randomized clinical trial, but has been studied in various retrospective reports. A key factor in the widespread utilization of this technique remains whether it can be determined to be safe, can improve outcomes, and is able to be performed by the wider neurosurgical community. In this systematic review, the questions of feasibility, safety, and efficacy of the supratotal resection technique will be examined in the literature regarding LGGs.

## 2. Materials and Methods

This systematic review followed guidelines concordant with PRISMA (Preferred Reporting Items for Systematic Reviews and Meta-Analyses) [18]. This checklist aims to improve the overall reporting ability of systematic reviews for randomized control trials (RCT) and observational studies. The study protocol was not registered with the Cochrane Database of Systematic Reviews.

### Search Strategy and Eligibility Criteria

One reviewer (M.D.G.) independently searched the PubMed, Medline, Ovid, CENTRAL (Cochrane Central Register of Controlled Trials), and Google Scholar databases on 17 January 2023 for studies evaluating overall survival, time to progression, seizure outcomes, and complications of supratotal resection (SupTR) of WHO-defined low-grade gliomas. A list of MeSH terms can be found in Supplemental Paragraph S1. The inclusion criteria were clinical outcome studies evaluating the supratotal resection of low-grade gliomas. The exclusion criteria were languages other than English, lack of full-text availability, inclusion of patients with only WHO-defined high-grade gliomas, and studies conducted on nonhumans. After an initial screening of titles/abstracts, the remaining papers were evaluated by full-text analysis by D.K. and M.D.G. The references examined were screened for eligibility and bias assessment utilizing internal quality and bias assessment within Covidence™ systematic review software. Citations were tracked and stored using Zotero citation software. Repeat articles were assessed using conditional formatting in Covidence™. After duplicates were removed, the abstracts and full texts were reviewed. Data were extracted using a standard template. The articles were reviewed for level of quality using the MINORS criteria [19]. In addition to taking the data as published, we performed a subanalysis where patients who were asymptomatic at diagnosis were compared with patients who had neurologic symptoms at diagnosis in terms of EOR, PFS, and OS.

## 3. Results

### 3.1. Search Results

**Figure 1:**
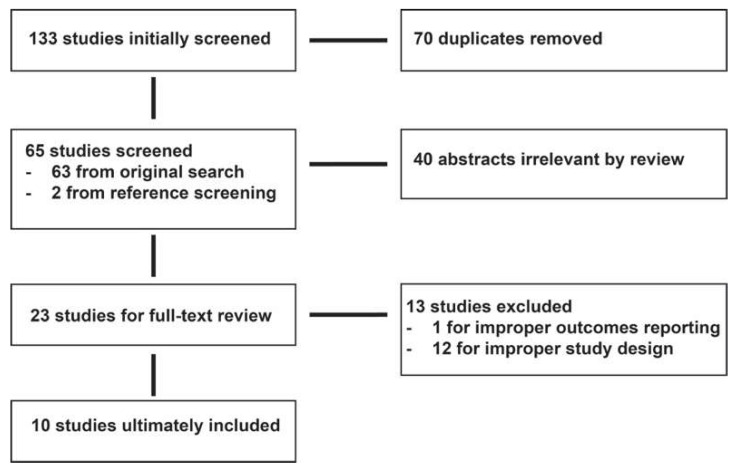
PRISMA flow diagram. Embase, Medline, Scopus, and Web of Science were searched for articles concerning supratotal resection in low-grade glioma, with reference lists also used as article sources. A total of 133 unique entries were considered for inclusion; 23 articles proceeded to full-text analysis, with 10 satisfying the criteria for review.

After initial database search, 133 studies were imported for screening. Of these, 70 duplicates were removed via screening in Zotero, leading to 63 studies being considered for abstract review. An additional 2 studies were considered from reference screening. From the abstracts, 42 studies were removed for being irrelevant or unrelated to the topic being studied, leaving 23 studies for full-text review. On full-text review, 12 articles were removed for study design flaws, and 1 was removed for improper outcomes reporting. A final flow diagram demonstrating the search process is shown in Figure 1. The 10 articles that were included in the final analysis were published in a range from 2011 to 2021. Study characteristics are reported in Table 1. Extended study findings are listed in Supplemental Table S1.

### 3.2. Overall Findings

Our final data extraction included 10 studies comprising 1301 total patients, of whom 377 (29.0%) underwent SupTR. There were 698 male (53.7%) and 603 (46.3%) female patients in total, with an age range of 18–75 years. Six of the studies were conducted solely in France, 2 solely in Italy, 1 in multiple countries (Italy, France, Canada, and the US), and 1 in India. Each of the studies reviewed focused on LGG and had at least a partial cohort of SupTR, with many studies including comparison cohorts of subtotal (STR) and gross total resections (GTR). All included studies had outcome measures reported that could be compared in this review.

The MINORS criteria [19] were utilized to evaluate nonrandomized studies. To qualify for inclusion and evaluation, noncomparative studies required a score of 10/14, while comparative studies required a score of 12/16. To assess the level of evidence, we additionally scored the papers based on the Oxford Center for Evidence Based Medicine Levels of Evidence Chart, with each paper scoring Level 3 or Level 4, indicating low to moderate quality of evidence [26]. It should be noted here that several of the studies have at least partial overlap between them in terms of both time frame for recruitment and likely patient population reported in the manuscripts. For the purposes of the review and because we did not perform significant additional analysis, we treated each study as an individual cohort, given that slightly different outcomes were reported for each published paper.

When reviewing the studies, special note was made regarding how each study defined “supratotal” resection. The papers associated with the group from France had a similar strategy across their publications: preoperatively, tumor volume was calculated using the largest diameter of FLAIR signal abnormality in the axial, sagittal, and coronal planes, and the ABC/2 method for ellipsoid was used to calculate tumor volume. Postoperatively at 3 months, the volume of residual tumor was measured in the same way. If there was no FLAIR abnormality and the 3-month resection cavity was larger than the preoperative tumor volume, the resection was deemed “supratotal” [12,20,21,22,23,25,27]. While it was often reported as a mean value, the individual values for 3-month resection cavity vs. preoperative tumor volume were not reported. The two papers from Italy utilized a similar method of comparing preoperative tumor volume with a postoperative resection cavity using MRI. This group calculated the preoperative tumor volume using BrainLab software for manual segmentation of the tumor. This volume was then compared with the resection cavity volume on a 2-month postoperative MRI. If the cavity was larger than the preoperative tumor volume (with noted adjustment for cavity collapse in deep or gravity-depending regions), it was deemed a supratotal resection [6,14]. In the manuscript featuring data from multiple centers, three of them (France, Canada, Italy) utilized the same methods as the French papers above, whereas the data from the USA were evaluated using BrainLab on the preoperative and immediately postoperative MRIs [25]. Finally, the publication from India used methods similar to the French group above, comparing preoperative tumor volume with the surgical cavity volume on 3-month postoperative MRIs [24].

### 3.3. Individual Studies

The first chronologically published study, Yordanova et al. [27] in 2011, featured 15 patients (mean age of 36.4 years, 8/15 male) with asymptomatic, “non-eloquent” gliomas who underwent SupTR along functional boundaries via awake craniotomy using the “asleep–awake–asleep” technique. The patients were compared with a control cohort of 29 patients who received GTR. In the supratotal cohort, only two had permanent postoperative deficits, and all patients had a Karnofsky Performance Status of 90 or better at 3 months postoperatively. When compared with the GTR cohort, fewer patients had anaplastic transformation (0 of 15 vs. 7 of 29, *p* = 0.037) and fewer required adjuvant therapies (1 of 15 vs. 10 of 29, *p* = 0.043) during the mean follow-up period of 35.7 months (6–135 months). There were no permanent deficits reported in the GTR group, and their KPS was not discussed. PFS for the SupTR group was reported as 38 months. OS was not specifically calculated, but no patients died during the study follow-up in either cohort.

A study by Lima and colleagues in 2015 [20] evaluated the risk of seizures in patients undergoing surgery for LGG. They prospectively evaluated 21 patients with a mean age of 35 years (range of 18–71 year), of whom 4 (19.0%) received supratotal resection, 10 GTR, and 7 STR. With a mean of 49-month follow-up, no permanent neurological deficits were seen, no patients died during the follow-up, and no patients in the SupTR or GTR groups had postoperative seizures. Two patients in the STR group developed seizures during the follow-up period. All patients were reported as fully functional in cognitive, social, and neurological domains. No patients receiving SupTR or GTR had adjuvant therapy, while all 7 in the STR group received adjuvant treatment. PFS was not reported, and OS was 100% at study end.

In 2016, Duffau et al. [21] presented a retrospective review of a prospective database of SupTR for incidentally found LGGs. The article was mostly a narrative description of long-term outcomes of SupTR, but had no control group or comparators, which detracts from the evidence presented. Of the 16 patients who underwent supratotal resection, 7 (43.8%) were male, and the average age was 41.3 years (range of 26–63). With an average of 132-month follow-up (range of 97–198), no permanent postoperative deficits were seen, no patients died, and 50% (8/16) eventually had tumor progression, for which 2 received re-resection. An official PFS was not calculated. Seizure control was excellent with 100% of the patients seizure-free after surgery. KPS was 90 or greater in all patients at 3 months postoperatively.

Lima et al. published another paper in 2017 [12] focusing on functional and neurological outcomes of SupTR for diffuse LGG in eloquent areas. All patients were asymptomatic at diagnosis, and all tumors were in the left hemisphere. Of 19 patients (8 males, 11 females) with a mean age of 32.8 years (range of 18–51), SupTR was achieved in 5 (26.3%), GTR in 5, STR in 7 (36.8%), and partial in 2 (10.5%). After surgery, no long-term seizure disorders (>6 months from surgery) were developed in the entire cohort. No permanent postoperative deficits were noted. During long-term follow-up (mean of 62.4 months (range of 21–231)), no patients that received GTR or SupTR had progression of their tumor and as such received no adjuvant therapy, while the partial and STR group had a median PFS of 65 months, and 7 of them received adjuvant treatments. No patients died during follow-up, and all had KPS of 100 at 6 months postoperatively. In this article, more evidence for the safety of SupTR was presented, but the total cohort of SupTR patients was very small, limiting generalizability.

In 2020, two studies from Ng et al. [22,23] were published which focused on return to work after resection of diffuse LGG in asymptomatic adults and neuropsychological assessments before and after awake surgery for LGG. The overall cohort in the return-to-work study contained 74 patients composed of 31 men and 43 women with a mean age of 35.7 years (range of 18–66) collected on a prospective basis, 21 (28.4%) of whom received supratotal resection, 22/74 GTR (29.7%), and 31/74 STR (41.9%). No permanent postoperative deficits were reported in the entire cohort. Of SupTR patients that were working at the onset of the study, 19 of 21 returned to work by the end of follow-up, versus 22/22 GTR and 31/31 STR patients. There were no statistically significant differences in return to work between the three subcohorts. At 5 years, 100% of the supratotal patients were alive, while four patients from the other cohorts died (3 GTR, 1 STR).

The neuropsychological assessment study retrospectively reviewed 47 patients (31 women, 16 men) with a mean age of 39.2 years (11.2 years SD) who underwent an asleep–awake–asleep protocol for tumor resection. This cohort included 12 SupTR, 19 STR, and 16 GTR patients, all of whom were asymptomatic and incidentally diagnosed. Preoperative neuropsychological assessments showed that 16 patients (34%) had “poor functioning” in at least one domain that was tested. Postoperatively, there was no significant difference between subgroups in terms of neurocognitive outcome, and no permanent postoperative deficits were seen. PFS was not reported, and no patients died during the mean follow-up of 33.0 months (range of 3–96). These two studies again focused on presenting evidence that SupTR is safe compared with GTR. They have larger cohorts, which increase the weight of the findings, but only show noninferiority in narrow outcome measures.

Two studies published by the same group in 2019 [6] and 2021 [14] contained partially overlapping cohorts (patients from 2011 to 2016 and 2009 to 2014, respectively) [6]. The study from 2019 focused on neuropsychological, quality-of-life, and oncologic outcomes after aggressive, functional boundary-based resections for suspected LGG. Of 449 patients included in the cohort, 145 (32.3%) of them received supratotal resection, 183 (40.8%) GTR, and 121 (26.9%) STR. In the SupTR subgroup, there were 77 men and 68 women (145 total), with a mean age of 37.9 years (median of 36.5 years), and 131 of these were found secondary to seizures, whereas only 14 (9.7%) were asymptomatic. After surgery, 1/145 SupTR patient (0.69%) had permanent neurologic deficits, similar to the GTR group (1/183, 0.55%). This was much lower (*p* < 0.001) than the 6.6% seen in the subtotal/partial resection cohort (n = 8/121). PFS and OS were not reported in this study. There were no differences between the SupTR and GTR groups in neuropsychological testing in any of the five domains evaluated. There were no differences between SupTR and GTR groups in return to work at 3 months or mean return-to-work time. Significantly more patients achieved epileptic control at 3-month follow-up in the SupTR cohort (55/60, 91.7%) than the GTR cohort (31/40, 77.5%) (*p* < 0.05).

The second article by Rossi et al. in 2021 [14] evaluated the association between supratotal resection and PFS, malignant transformation, and OS in LGG. The study cohort included 319 patients, 306 of whom presented with seizures at diagnosis (95.9%). All underwent functional boundary-guided surgery for IDH-mutant LGG with the goal of supratotal resection, which was achieved in 110/319 (35%). Permanent postoperative deficits occurred in none of the supratotal resection cohort and 6/209 (2.9%) of the remaining patients. With a median follow-up of 6.8 years, 190/319 (59.6%) of the patients had progression, with a median PFS of 4.7 years (95% CI 4–5.3 years). Lower EOR was strongly associated with recurrence, as only 5.4% of the SupTR group recurred (6/110, PFS = not reached), while 82.4% of GTR (103/125, PFS = 46 months), 95.5% of STR (71/74, PFS = 29 months), and 100% of partial resections (10/10, PFS = 27.5 months) recurred. The members of the SupTR group also had a lower amount of malignant transformation (of those who recurred, 16.6% SupTR, 67.0% GTR, 69.1% STR, 40% partial recurred as higher grade) and were placed on adjuvant therapies at a lower rate. Finally, OS was evaluated with regard to EOR, and at 45 months, 70% of partial, 83.8% of STR, 83.2% of GTR, and 100% of SupTR patients remained alive. These two articles provide strong statistical backing from large cohorts on the outcomes of SupTR and the factors that may contribute to the achieving a greater EOR.

Goel et al. [24] published a large series of “en masse” resections of LGGs affecting only the short arcuate fibers in 2021, detailing 74 patients (mean age of 33, range of 21 to 55) identified retrospectively. Of the 46 males and 28 females in the study, 25 (33.8%) had SupTR, 37 (50%) received GTR, and 12 (16.2%) had STR. These patients were all symptomatic on presentation. Importantly, it must be stated that the patients’ tumors all had the precondition of being located in “short arcuate fibers”, meaning that tumors located in the insula, deep white matter tracts, and other diffuse connecting areas were not included. Postoperatively, all patients had improvement in seizure seismology, none had changes in their neurocognitive evaluations, and no permanent postoperative deficits were reported. During follow-up (average of 30 months, range of 11–52 months), there were no recurrences or reported complications in the SupTR group as compared with the GTR group, which did have one recurrence. None of the STR patients had growth of their residuals during follow-up. Moreover, the SupTR group had no patients receiving adjuvant therapy (nor did the GTR), but all 12 subtotal resection patients underwent both chemotherapy and radiotherapy. Goel et al. provide a narrative description of SupTR outcomes in a non-Western medical center, which increases the generalizability of the overall findings of the SupTR for LGG literature.

Lastly, for the reviewed articles, a paper by Ius et al. (2022) [25] reviewed 267 patients from four different centers in Montpellier, France; Udine, Italy; Calgary, AB, Canada; and San Francisco, CA, USA. The stated goal of this study was to identify factors that influenced outcome and survival for incidental LGG patients treated with early surgery. Of 267 total patients, 155 (58.1%) were female, 112 (41.9%) were male, and 24 (9.0%) underwent SupTR. The mean age of this cohort was 39.2 years (18–71 range). At 100 months, OS was 100% for patients in the SupTR group compared with 98% in the GTR group and 87.5% in the STR group. In univariate regression analysis, SupTR or GTR were significantly associated with reduced risk of death compared with STR, but this effect disappeared in the multivariable analysis. In their regression analysis for tumor recurrence, SupTR or GTR were not statistically better than STR. Again, this study provides a weighty cohort of patients and strong statistical evaluation, but can only conclude that SupTR was not inferior to GTR.

To examine whether there were data differences between symptomatic and asymptomatic patients, we grouped the studies that were specific to asymptomatic patients [12,20,21,22,23,25] and those specific to symptomatic patients [6,14,24,27] and compared the reported outcomes including permanent deficits, OS, and PFS as reported for the supratotal resection patients. In the asymptomatic patients, a total of 113/444 published cases (25.5%) were able to be supratotally resected, with almost no permanent postsurgical deficits (3.1% for all cases in Ius et al., 0% for other studies). All studies reported 100% survival at study end for asymptomatic SupTR patients. For symptomatic patients, a total of 295/857 patients (34.4%) were supratotally resected, again with very low rates of permanent deficits (3/295 patients, 1.0%) in SupTR patients. Only three of four symptomatic studies reported OS; two reported 100% OS at study end [24,27], and one reported 94% at 92 months for their SupTR group [6].

## 4. Discussion

There remains controversy regarding supratotal resection of gliomas, partially because there are little high-quality clinical data published on the topic. Recent clinical studies have investigated the role of supratotal resection in further improving clinical outcomes, such as progression-free survival, overall survival, and neurologic morbidities, compared with gross total resection. The primary argument for supratotal resection of LGG is that a more aggressive resection can achieve a greater margin of resection, reducing the risk of malignant transformation and improving clinical outcomes [13,21,27,28,29]. However, supratotal resection may increase risks of neurologic complications relative to gross total resection through the removal of potentially functional brain around the tumor [13,22,23,30]. Thus, the clinical impact of supratotal surgical resection remains controversial.

This review evaluated 10 studies that specifically reported patients who underwent supratotal resection of low-grade glioma. In many of the studies, the authors measured the volume of the tumor preoperatively and compared it with the postoperative surgical cavity on 3-month MRI, confirming the supratotal extent of resection [6,12,14,20,21,22,23,25,27]. This allowed separate outcomes reporting from the gross total resection and subtotal/partial resection patients that were also analyzed in each of the studies. In all of the studies, at least a proportion of the cohort underwent surgical resection that relied upon using functional mapping via electrical pulses in order to determine whether an area was functional or not. This was performed under an asleep–awake–asleep protocol or general anesthesia for all published cases. The use of intraoperative MRI was variable across the studies, which did not affect outcome reporting, but it could have made the percent supratotal achievement better for groups that used it, and it is considered an irreplaceable surgical tool in the armament of many glioma surgeons. Intraoperative MRI could have provided confirmation on the day of surgery that a supratotal resection was achieved, as it is not subject to the forces of healing and brain shift over 3 months. Moreover, differences in MRI follow-up and the definition of “supratotal” differed significantly between papers, as mentioned previously. This is a highly important part of the argument surrounding SupTR, as definitions must be consistent among practitioners to come to adequate conclusions surrounding the outcomes of the technique. By narrowing and strictly defining what “supratotal” resection means and how it is documented, objective and directly comparable evidence can be built up through clinical trials. This must be done to ensure that true supratotal resections are compared with other types in the future.

Another set of key differences between papers was the breakdown of patient groups, study design, and the preoperative symptomatology. There was no consistent study design or patient characteristic, nor was there any degree of randomization or blinding. Among the included studies, some included comparisons with other extents of resection groups, such as GTR and STR, whereas other studies presented supratotal resection cohorts without comparisons. Data analyzed included retrospective reviews and retrospective analyses of prospective data sets. These study design differences complicate comparisons. Additionally, retrospective cohort and case series have inherent biases, including selection and treatment biases, which can alter the quality of the data. This range of study designs leads to a body of evidence from which it is difficult to draw generalizable conclusions, and one that is likely not representative of a true “real world” sample of all-comers. However, given the lack of randomized trials on this topic, an analysis of the available literature can speak to trends in the data.

Of particular importance for the interpretation of results is that the symptomatology of the patients was significantly mixed. Four of the studies focused exclusively on asymptomatic or “incidental” LGGs, which represent only a small total of the overall LGG population. These two groups of LGGs are considered distinct by the neurosurgical literature, as incidental gliomas remain controversial in terms of treatment planning, though current practice has evolved such that early surgery is currently favored [5,30,31]. Our regrouping of SupTR patients based on preoperative symptomatology did not demonstrate wide differences in the percentage of patients receiving SupTR, permanent neurological deficits, or OS. Based on this, available data suggest that preoperative symptomatology is not impactful on the ability to achieve supratotal resection and does not affect postoperative outcome.

Supratotal resection is being adopted more widely as evidenced by recent literature, but a number of flaws in the current data merit discussion. Despite the increased adoption of supratotal resection (346 reported patients from 2019 to 2023 compared with 31 from 2011 to 2017) [11], this technique remains sparsely reported in the literature. Another weakness of the data is that over half of the presently reviewed cases were associated with papers authored or coauthored by a single group [12,20,21,22,23,25,27]. This group has published multiple manuscripts regarding the surgical strategy, patient selection, outcomes, and approach to recurrent LGG, and readers are directed to their work for a more comprehensive discussion [32,33,34,35,36]. Although this speaks to their experience and expertise on this topic, comparable data from a wider range of authors would help confirm the reproducibility of the clinical results. In terms of patient cohorts, the overall population among the clinical studies reviewed here was homogeneous, mainly European or North American from large tertiary referral centers in major metropolitan areas. Moreover, the centers publishing manuscripts on supratotal resection of LGG represent high-volume glioma centers and thus contain the infrastructure to support these surgeries in a way that may not be representative of most hospitals. Because of these characteristics, the studies included in the review represent a relatively narrow proportion of the total LGG patient pool, selected because they were good candidates for case series or cohort studies. Each of these points may detract from the potential to generalize these results to patients and clinical situations that differ.

The overall the quality of evidence presented in the 10 studies here is low. Using Oxford Center for EBM Levels of Evidence, these clinical studies were consistent with Level 3 and Level 4 data, consisting mostly of retrospective, unblinded cohort studies or case series from a single institution [26]. Some data were consistently reported throughout the group of studies and provide argument in support of the safety and efficacy of supratotal resection. These data include improved OS [12,22,25], improved PFS [12], reduced need for adjuvant therapy [12,20,22,24,27], and improved postoperative seizure control [6,22]. However, there were inconsistencies in study objectives, leading to differences in outcome reporting.

Regarding overall and progression-free survival, the outcomes were reported slightly differently across the manuscripts included. Although two studies explicitly compared OS [22,25], neither was specifically designed to prove that SupTR was beneficial in OS. Other studies often reported OS but either had too short of follow-up or too few patients to make comparisons or report meaningful percentages, and several just reported that no patients died during the study. Many of the studies reported PFS, but Lima et al. in 2017 [12] was the only one where the SupTR group had an improvement over another group. For the purposes of this review, these data were not ideal as SupTR and GTR were combined (no recurrences) and compared with the STR/partial group, which had a median PFS of 65 months. Thus, no specific conclusion regarding the benefit of SupTR in terms of PFS could be made. Additionally, in the other studies, there was evidence that in spite of supratotal resection, there can be recurrences, such as the Duffau 2016 report of long-term follow-up of only SupTR patients [21]. This paper reported that 8 of 16 SupTR patients (50%) had recurrences, and 2 underwent reoperation over a mean of 132-month follow-up period. Given these caveats, the current data suggest that SupTR does not convincingly improve either OS or PFS [29,37].

To improve PFS and OS, patients considered high risk may receive adjuvant therapy with chemotherapy, radiation, or both. This generally includes any patient with subtotal or partial resections. Most of the studies reported adjuvant treatment decisions in their EOR cohorts. There were large differences between groups reported in several studies, with greater EOR leading to lower reported use of adjuvant therapy in the study follow-up periods [12,20,24,27]. These data do not point strongly at SupTR as the reason for reduced adjuvant therapy usage; instead, it suggests that adjuvant therapy is employed when SupTR (and by extension, GTR) is not able to be completed due to concerns regarding potential neurologic morbidity. Permanent postoperative neurological deficits can affect long-term survival [35,38,39,40], and surgeons will typically prioritize neurologic function over the extent of resection and leave behind tumor that maps to eloquent areas. This indirectly speaks to the feasibility of supratotal resection in that only select patients can be reasonably expected to be candidates for SupTR safely with preserved function.

Regarding postoperative neurological outcomes, the most significant support for supratotal resection comes from the data surrounding seizure control. Rossi and colleagues reported significant differences in the number of SupTR patients (55/60, 91.7%) vs. GTR patients (31/40, 77.5%) who were able to achieve epileptic control at 3-month follow-up (*p* < 0.05). Almost all of the other studies reported a very low rate of seizures in their total cohorts, indicating that SupTR did not increase the seizure risk over GTR or STR. Additionally, nearly all patients were able to reduce their AED regimens over 3–12 months postoperatively. For studies reporting SupTR for incidental LGG, the focus of outcome reporting was KPS at 3 months or return to work/normal life, likely because the authors aimed to show that SupTR was not inherently riskier than GTR. In this realm, it can be summarized that SupTR is not causing permanent harm to patients, as the vast majority return to work and enjoy a normal social life postoperatively. This is true notwithstanding that each study reported a sizeable percentage of patients who had identifiable immediately postoperative neurological deficits but very low numbers (often zero percent) of patient with permanent postoperative deficits. This echoes what has been published in the literature in both low- and high-grade gliomas, that transient deficits, while troublesome after initial functional boundary-guided resection, do not often lead to permanent postoperative deficits [41,42,43,44]. Together these findings suggest that despite initial worsening when resecting supratotally along functional boundaries, patients do recover their function, and the procedure is not inherently riskier than GTR.

## 5. Conclusions

Overall, the data present a trend toward a cautious recommendation to aim for supratotal, functional boundary-guided surgery when resecting LGGs in either asymptomatic or symptomatic patients. In the available data, patients who receive SupTR continue working, do not require adjuvant therapy, enjoy reduced seizure burden, and have a good quality of life, similar to or better than patients receiving GTR or STR. One possible conclusion is that SupTR is noninferior to GTR, but there is no evidence to suggest that SupTR should be achieved at the cost of undermining neurological function, echoing the literature detailing supratotal resection of high-grade gliomas [8,11,38,39]. Although a few studies reported improved OS and PFS, the study designs were not proper to assert a difference in these outcome measures and, as such, cannot be used to defend the superiority of this approach. Additionally, since GTR and SupTR groups were combined in many of the studies to report outcomes, it is difficult to identify the advantages of SupTR in these studies. Additionally, many studies lacked long-enough follow-up to accurately measure PFS and OS in the SupTR and GTR cohorts. Given the noninferiority of SupTR in terms of neurologic morbidity and short- and intermediate-term outcomes, additional clinical studies are warranted to build the strength of evidence as the neurosurgical and neuro-oncology communities await long-term clinical outcome data necessary to distinguish possible survival benefits of SupTR over GTR in LGG.

This systematic review is limited by the fact that there is a paucity of overall data published, and that the studies available are inherently of low quality of evidence because of study design. Improper control groups, lack of control groups, lack of randomization, and other issues plague the current supratotal resection literature, and this issue is ideally remedied with high-quality clinical studies specifically comparing gross total and supratotal resection strategies in both symptomatic and asymptomatic patients. This approach must be considered in order to truly answer whether SupTR improves PFS, OS, and overall outcome of surgery.

## Figures and Tables

**Table 1:** Key characteristics of included studies.

Study ID	Study Country	Study Design	Start Date	End Date	Total Number of Patients	Supratotal Resection Sample	% Male	Age at Resection	Permanent Neurological Deficits (N, %)	Progression-Free Survival	Overall Survival
Yordanova et al. (2011) [13]	France	Case series	1998	2010	15	100.00%	53.3	36.4 (24–59)	2, 13.3%	73.3% at 38 months	100% at study end
Lima et al. (2015) [20]	France	Case series	1998	2012	21	19.0% (4/21)	28.57	35 (18–57)	0, 0%	100% at study end	100% at study end
Duffau et al. (2016) [21]	France	Cohort study	1998	2007	16	100.00%	43.75	41.3 (26–63)	0, 0%	50% relapse rate (avg 70 months)	100% at study end
Lima et al. (2017) [12]	France	Two-center prospective study	1998/2010	2010/2013	19	26.3% (5/19)	42.1	31.2 (19–51)	0, 0%	100% at study end	100% at study end
Rossi et al. (2019) [6]	Italy	Case series	2011	2016	449	32% (145/449)	53.1	37.9 (median 36.5)	1, 0.69% (SupTR group)	Not reported	Not reported
Ng et al. (2020) [22]	France	Case series	1998	2017	74	28% (21/74)	41.89	35.7 (18–66)	0, 0%	Not reported	100% at 5 years
Ng et al. (2020) [23]	France	Case series	2011	2019	47	26% (12/47)	34.04	39.2 +/− 11.3	0, 0%	Not reported	100% at study end
Goel et al. (2021) [24]	India	Cohort study	2016	2019	74	34% (25/74)	62.16	33 (21–55)	0, 0%	98.7% at 2 years	100% at study end
Rossi et al. (2021) [14]	Italy	Case series	2009	2014	319	35% (110/319)	61.1	38.9 (18–75)	6, 1.9%	94% at 92 months (SupTR group)	100% at 80 months (SupTR group)
Ius et al. (2022) [25]	USA, Canada, France, and Italy	Four Center Retrospective Review	1998	2019	267	9% (24/267)	41.9	39.2 (18–71)	8, 3.1%	Not reported	100% at 100 months (SupTR)

## Data Availability

All data was obtained from the referenced papers herein.

## Supplementary Materials

The following supporting information can be downloaded at: https://www.mdpi.com/article/10.3390/cancers15092493/s1, Paragraph S1: MESH Terms; Table S1: Extended Findings of Included Studies.
