# Supplementary material for: Supratotal Surgical Resection for Low-Grade Glioma: A Systematic Review
Source: Cancers (Basel). 2023 Apr 26;15(9):2493. doi: 10.3390/cancers15092493 (PMC10177219; doi:10.3390/cancers15092493)
Supplement: Supplementary file 1 [file cancers-15-02493-s001.zip › cancers-2344469-supplementary.pdf]

Table S1. Key Characteristics of Included Studies; USA = United States of America.

Study ID	Study Country	Study Design	Start Date	End Date	Total Number of Patients	SupraTotal Resection Sample	% Male	Age at Resection	Permanent Neurological Deficits (N,%)	Progression-Free Survival	Overall Survival
Yordanova et al. (2011) [13]	France	Case series	1998	2010	15	100.00%	53.3	36.4 [24-59]	2, 13.3%	73.3% at 38 months	100% at study end
Lima et al. (2015) [20]	France	Case series	1998	2012	21	19.0% (4/21)	28.57	35 [18-57]	0, 0%	100% at study end	100% at study end
Duffau et al. (2016) [21]	France	Cohort study	1998	2007	16	100.00%	43.75	41.3 [26-63]	0, 0%	50% relapse rate (avg 70 months)	100% at study end
Lima et al. (2017) [12]	France	Two Center Prospective Study	1998/ 2010	2010/ 2013	19	100.00%	42.1	31.2 [19-51]	0, 0%	100% at study end	100% at study end
Rossi et al. (2019) [6]	Italy	Case series	2011	2016	449	32% (145/449)	53.1	37.9 (median 36.5)	1, 0.69% (SupTR group)	Not reported	Not reported
Ng et al. (2020) [22]	France	Case series	1998	2017	74	28% (21/74)	41.89	35.7 [18-66]	0, 0%	Not reported	100% at 5 years
Ng et al. (2020) [22]	France	Case series	2011	2019	47	26% (12/47)	34.04	39.2 +/- 11.3	0, 0%	Not reported	100% at study end
Goel et al. (2021) [24]	India	Cohort study	2016	2019	74	34% (25/74)	62.16	33 [21-55]	0, 0%	98.7% at 2 years	100% at study end
Rossi et al. (2021) [14]	Italy	Case series	2009	2014	319	35% (110/319)	61.1	38.9 (18-75)	6, 1.9%	94% at 92 months (SupTR group)	100% at 80 months (SupTR group)
Ius et al. (2022) [25]	USA, Canada, France, and Italy	Four Center Retrospective Review	1998	2019	267	9% (24/267)	41.9	39.2 [18-71]	8, 3.1%	Not reported	100% at 100 months (SupTR)

Table S2. Extended findings of included studies. AED = anti-epileptic drug; KPS = Karnofsky performance scale; SMA = supplementary motor area; SupTR = supratotal resection; STR = subtotal resection.

Study ID	Study Country	Study Design	Total Number of Patients	SupraTotal Resection Sample	Transient Neurological Deficits (N, %)	Types of Transient Deficits	Recovery by 3 Months	KPS at 3 months	Seizure Control Postoperatively	Adjuvant Chemotherapy (N, %)	Adjuvant Radiotherapy (N,%)
Yordanova et al. (2011) [13]	France	Case series	15	100.00%	9, 60%	SMA Syndrome (2/9), Language deficit (7/9)	100%	>90 for 14/15 patients, 80 for 1 patient	Improved in all patients	0/15, 0%	1/15, 6.7%
Lima et al. (2015) [20]	France	Case series	21	19% (4/21)	7/21, 33%	SMA Syndrome (2/7), Language deficit (5/7)	100%	100 for all cases	One postoperative seizure, all AEDs stopped by 8 months postop	6/21, 28.6%	1/21, 4.8%
Duffau et al. (2016) [21]	France	Cohort study	16	100.00%	9/16, 56.2%	SMA syndrome (2/9), Language deficit (9/9)	100%	90-100 for all cases	Improved in all patients	3/16, 18.8%	2/16, 12.5%
Lima et al. (2017) [12]	France	Two Center Prospective Study	19	26.3% (5/19)	10/19, 52.6%	SMA syndrome (3/10), Language deficit (7/10)	100% (at 6 months)	100 for all cases	Improved in all patients	4/19, 21.1%	2/19, 10.5%
Rossi et al. (2019) [6]	Italy	Case series	449	32% (145/449)	399/449 total (88.9%), 127/145 SupTR (87.6%)	Speech changes, motor delays, not otherwise specified	99.3% (144/145)	90-100 for all cases	91.7% (51/60 patients analyzed)	N/A	N/A
Ng et al. (2020) [22]	France	Case series	74	28% (21/74)	N/A	N/A	100%	N/A	Long-term seizures in 4/74, preventing or delaying return to work	24/74 (32.4%	7/74 (9.5%)
Ng et al. (2020) [22]	France	Case series	47	26% (12/47)	26/47, 55.3%	SMA syndrome (3/26), Language deficit (17/26), hemiparesis (3/26),	100%	N/A	3/47 (6.4%) with continued seizures 12 months postop	11/47 (23.4%) on avg 30 months postop	5/47 (10.6%) on avg 32

						visuospatial deficits (3/26)					months postop
Goel et al. (2021) [24]	India	Cohort study	74	34% (25/74)	6/74 (8.1%	SMA syndrome (2/6), Motor weakness (3/6), Language deficit (1/6)	100%	N/A	Improved in all patients	12/74 (16.2%), all STR patients	12/74 (16.2%), all STR patients
Rossi et al. (2021) [14]	Italy	Case series	319	35% (110/319)	293/319 (91.8%) total cohort, 100/110 (90.9%) SupTR cohort	N/A	100%	N/A	N/A	10/110 SupTR (9.1%)	5/110 SupTR (4.5%)
Ius et al. (2022) [25]	USA, Canada, France, and Italy	Four Center Retrospective Review	267	9% (24/267)	77/267 (32.6%)	SMA syndrome (13/77), Language deficit (42/77), Motor deficit (7/77), Visual field deficit (4/77), Facial droop (4/77), Spatial neglect (3/77), Other (4/77)	251/259 (96.9%)	N/A	N/A	32/267 (11.98%)	6/267 (2.2%)
